# The impact of care-recipient relationship type on mental health burden of caregivers for schizophrenia patients: evidence from Beijing, China

**DOI:** 10.3389/fpsyg.2024.1402159

**Published:** 2024-06-14

**Authors:** Yi Zhu, Margaret Xi Can Yin

**Affiliations:** ^1^School of Public Administration, Northwest University, Xi’an, China; ^2^School of Humanities, Southeast University, Nanjing, China

**Keywords:** care-recipient relationship type, caregivers’ mental health burden, schizophrenia patients, China, influencing factors

## Abstract

**Objective:**

To examine the impact of care-recipient relationship type on mental health burden of caregivers for schizophrenia patients in China, elucidating the underlying mechanisms.

**Methods:**

A cross-sectional study was conducted using face-to-face surveys administered to caregivers of patients with schizophrenia in selected communities in Beijing, China. 1,853 samples’ data was used. Descriptive statistics, logistic regression models and Sheaf coefficient method were employed to analyze the data.

**Results:**

The mental health burden experienced by caregivers of schizophrenia patients has reached a high level, with 66.9% reporting a moderate or severe impact from their caregiving responsibilities. Parents and spouses were the primary providers of care for schizophrenia patients in China. Parent caregivers experienced greater suffering in their caregiving role compared to spouse caregivers, whereas the difference between child caregivers and spouse caregivers was not significant. The factors influencing caregiver’s mental health burden vary according to the type of relationship with the care-recipient. For parent caregivers, the mental health burden primarily stems from personal conditions of schizophrenia patients, while for spouse or child caregivers, it mainly arises from family economic conditions.

**Conclusion:**

This study reveals that caregivers having different types of care-recipient relationship with schizophrenia patients experience significantly different mental health burdens in Beijing, China, and major influencing factors are distinct according to different care-recipient relationship types.

## 1 Introduction

Schizophrenia is a debilitating mental illness, which compromises cognitive functions, disrupts interpersonal dynamics, and engenders emotional, social, and economic hardships (Vigo et al., 2016; Onitsuka et al., 2022). Caregivers hold a crucial role in the lives of patients with schizophrenia. They provide direct care, assistance with activities of daily living, and emotional, social, and financial support to patients with schizophrenia, but their mental health can often be overlooked by the public (Prasad et al., 2024). With the deinstitutionalization movement in the West, 25 to 50% of individuals with schizophrenia receive care within the community or from their families, with this figure rising to as high as 70% in Asian countries (Chan, 2011). Caring for patients with schizophrenia poses unique challenges due to the multifaceted nature of the illness (Juntapim and Nuntaboot, 2018). Caregivers of patients with persistent symptoms often feel overwhelmed, stressed, drained, burdened, frustrated, or angry (Kamil and Velligan, 2019). The burden of caregiving for family members is characterized as “a psychological state resulting from a combination of physical labor, emotional and social pressures, and economic constraints associated with caring for the patients” (Dillehay and Sandys, 1990; Chen et al., 2019).

Family or informal caregiving burden is a global challenge (Hsiao and Tsai, 2015). Providing care to family members may bring heavy burden for caregivers which are often overlooked. Hoenig and Hamilton (1966) have categorized the caregiving burden into two distinct types for family members: objective burden and subjective burden. Objective burden refers to the impacts on health, financial cost, or daily life chores, while subjective burden pertains to the psychological pressure perceived by the caregiver from the care activities (Awad and Voruganti, 2008). Caqueo-Urízar et al. (2009) summarized the quality of life of caregivers and found that their health deteriorates, leading to stress, anxiety, and depression during the caregiving process. For caregivers of schizophrenia patients, the increased duration of illness and care, severe or persistent schizophrenia symptoms, criticism of the care recipient, financial burden, and patient disability intensify their caregiving burden (Kamil and Velligan, 2019).

Caregiver burden is significantly influenced by the type of relationship with the care recipient (Lai et al., 2022). Caregiver’s role is often perceived primarily in terms of the effects of caregiving (Schulz et al., 2020), especially among family caregivers. For instance, there were significant differences in physiological function, bodily pain, general health, vitality, social function, mental health, each dimension of social support, and quality of life among caregivers having different relationships with the patients (Li et al., 2017). Providing care within families can negatively impact the relationship quality between caregiver and recipient. Extensive literature demonstrate that, in the case of spousal caregivers of individuals with dementia, behavioral symptoms such as negative emotional expression pose challenges for caregivers, leading to a decline in relationship satisfaction and a reduction in emotional and physical intimacy (Simonelli et al., 2008; Ascher et al., 2010). Among spousal caregivers of older adults with multiple chronic conditions, both caregivers and care recipients experience lowered relationship satisfaction when facing depressive symptoms and lowered self-reported health. This effect is particularly pronounced for care recipients experiencing high levels of depression (Monin and Schulz, 2009). However, these aspects are relatively underexplored regarding to caregivers for patients with schizophrenia.

Although the influence of care-recipient relationship type on mental health burden of caregivers has received some attention, several issues persist. Firstly, care-recipients are often the elderly, adolescents, or other minority groups, such as cancer patients, meaning that relatively few studies have focused on caregivers of schizophrenia patients (Ferraris et al., 2022; Chen and Li, 2023). Peng et al. (2023) used a Chinese version of the Family Questionnaire and found that in China, parents or spouses scored higher in emotional over involvement with schizophrenia patients compared to siblings, but no significant difference existed between fathers, mothers, or spouses. Secondly, most existing studies concentrated on the characteristics or correlations of factors influencing caregivers’ quality of life, lacking research on specific issues such as the impact of caregiver’s mental health burden (Srivastava, 2005; Chua et al., 2023). Additionally, the number of research interviewees or samples typically does not exceed 500 (Reinhard and Horwitz, 1995; Knock et al., 2011; Chen et al., 2019). Therefore, exploring the influence of care-recipient relationship type on mental health burden of caregivers of schizophrenia patients using a larger sample size is of great significance.

The care-recipient relationship type can be categorized into four main types: parents, spouses, adult children, or other relationships (Kamil and Velligan, 2019). In the case of parental caregivers, Cook’s study in the US found that both parents, particularly mothers, of patients with mental illness experienced high levels of emotional distress, including anxiety, depression, fear, and emotional drain, with mothers often reporting higher degrees compared to fathers (Cook, 1988). Parental caregivers may experience increased stress due to concerns about the safety of their child and others, especially when the mental condition of the patient is unstable. Additionally, in rural China, parental caregivers may face stigma related to mental illness, leading to discrimination by neighbors (Peng et al., 2019).

For spousal caregivers and child caregivers, whether wives or husbands, the risk is high as they often bear the burden of caregiving without additional support (Cohen et al., 1993). Previous studies suggested that spousal caregivers experienced heightened stress and may also face significant pressures regarding work and household responsibilities if their partners were unable to contribute to family financial obligations (Peng et al., 2019). Li and Dai (2019) found that in China, spouses were often assumed to be responsible of caregiving, even when living with their adult children. Research from South Korea also indicated that spousal caregivers perceived more burden overall compared to adult child caregivers, while sons perceived less burden than daughters in most areas (Hong and Kim, 2008). Using Italian data, Rinaldi, Spazzafumo (Rinaldi et al., 2005) found that compared to friends or other relative caregivers, adult child or spousal caregivers of dementia patients have a higher risk of burden and distress.

For other relative caregivers, such as siblings, caregiving can be particularly stressful as it may not align with normative caregiving roles (Avioli, 1989). Yeager et al. (2010) found that family members reported more burden than other types of caregivers of patients with Alzheimer’s disease, with caregiving children reporting significantly more burden than spouses or other relatives. These situations might be different for caregivers of schizophrenia patients.

The influencing mechanism of care-recipient relationship type on caregiver’s mental health burden remains to be studied. Previous literature has clearly demonstrated that caregiving is burdensome and psychologically distressing to family members, but the causal mechanism of this distress is not clear (Schulz et al., 1995). Caregiver’s mental health burden mainly stems from care recipient’s personal abilities, caregiver’s own abilities, family economic conditions, as well as the availability of public services in the community. As the care recipients’ health condition progressively declines, family caregivers may encounter difficulties in adapting or modifying their caregiving strategies, leading to a significant high level of caregiving burden (Papastavrou et al., 2007). Specifically, an old age, a low socioeconomic status, and a low education level are significantly associated with higher levels of caregiver burden (Schölzel-Dorenbos et al., 2009). Furthermore, dementia severity, presence of behavioral disturbances, extent of personality change, and presence of schizophrenia symptoms are also significant factors contributing to the increased level of caregiving burden (Chiao et al., 2015).

In summary, caregivers of schizophrenia patients bear a significant mental health burden and require more attention. Previous studies have focused more on nursing stress among patients with various physical illnesses and have given insufficient attention to caregivers of patients with mental illness, particularly the influence of relationship type on mental health burden of caregivers of patients with mental illness and its underlying mechanisms. Therefore, using a cross-sectional citywide survey data from Beijing, this research aims to investigate the impact of different care-recipient relationship types on mental health burden of schizophrenia patients in China and its influencing mechanisms.

## 2 Materials and methods

### 2.1 Data sources and sample collection

This study used data from a cross-sectional survey conducted among registered patients with schizophrenia in Beijing communities. Face-to-face and telephone interviews were employed during the survey process, utilizing a multi-stage stratified sampling procedure. The survey was jointly conducted by Renmin University and Capital Medical University in China in 2019. A total of 2,994 survey samples were collected from 16 districts of Beijing, with sample sizes ranging from 80 to 500 per district based on population proportions. After excluding records with missing variables, the effective sample size for the analysis was 1,853. The survey was conducted anonymously and did not include any sensitive information. Interviewees participated voluntarily and provided informed consent. Ethical approval was obtained from the Medical Ethics Committee of Capital Medical University in China (Zhu et al., 2021). The data have several advantages: firstly, it was collected in Beijing, the capital city of China, thus representing one of the most developed areas of the country; secondly, the large sample size and reasonable sampling method enhance the credibility of the conclusions drawn from the study.

### 2.2 Measurements

#### 2.2.1 Dependent variable

Caregiver’s mental health burden. The measurement is derived from the Family Burden Interview Schedule (FBIS) proposed by Pai and Kapur (1981), a widely used tool known for its reliability and validity (Karambelas et al., 2022). The Family Burden Interview Schedule (FBIS) was translated into Chinese, comprising 24 items across six dimensions. These dimensions include financial burden (6 items), disruption of family routine and leisure (9 items), disruption of family interactions (5 items), and the effects on the physical health (2 items) and mental health (2 items) of relatives. Given the focus of this study on caregivers’ mental health burden, we primarily selected the measurement instrument to assess the emotional impact of schizophrenia patients on caregivers. Specifically, the question asked, “whether or not the family members’ emotion had been impacted (depressive symptoms, crying or angry).” Subjects indicated the extent of impact on a 3-point scale ranging from no impact to moderate impact to severe impact. We recoded the variable into a binary variable, with no impact recoded as 0 and moderate or severe impact recoded as 1.

#### 2.2.2 Independent variable

Caregiver-recipient relationship type. The measurement question is, “What is your relationship with the patient?” The options include father, mother, father-in-law, mother-in-law, spouse, sibling, son, daughter, grandchild, other relatives, friends, colleague, and others. We recoded the options into four distinct groups: parents, spouse, children, and others. In the empirical analysis, we treated the spousal caregiver group as the reference category.

#### 2.2.3 Control variables

Firstly, caregiver’s personal characteristics, involving their education level and self-rated caregiving ability. Education level was measured as a continuous variable, while self-rated caregiving ability was measured as a dichotomous variable. Secondly, care recipients’ characteristics include gender, age, disability certification status, treatment status, and duration of schizophrenia for the care recipients. Thirdly, family economic conditions and community factors: regarding family conditions, the survey considered the living arrangements of caregivers and patients, family income level, expenditure on medication and healthcare services, and medical insurance coverage. As for community factors, the survey inquired whether the community provided free healthcare services. Detailed results are presented in Table 1.

**Table 1:** Descriptive results (*N* = 1853).

Variable	Share/Mean(SD)	Min	Max	Note
Caregiver’s Mental Health Burden				
Moderate or severe impacted	66.9%			(No Impact = 0)
	1.88(0.73)	1	3	Continuous variable
Care-recipient relationship type				
Parents	31.0%			
Spouses	35.4%			
Children	13.3%			
Other	20.3%			
Care givers				
Education Year	9.96(4.32)	1	19	
Self-Rated Ability (Strong = 1)	85.59%	0	1	(weak = 0)
Care recipients				
Gender (Female = 1)	53.86%	0	1	(Male = 0)
Age/10	5.93(1.22)	2.1	9.7	
Disability Certif. (Certificated = 1)	65.08%	0	1	(Not Certif. = 0)
Under Treatment (Yes = 1)	93.42%	0	1	(No = 0)
Time/10	0.93(0.25)	0	1	Duration of Schizophrenia
Time/10 Square	1.36(1.35)	0	6.7	
Family and community factors				
Living together (Yes = 1)	84.62%	0	1	(No = 0)
Family income (log)	9.01(1.10)	2.30	14.19	ln (income)
Consumption on Med.(log)	0.28(0.25)	0	1	Cons. on Med.
Market Care Service (Yes = 1)	3.78%	0	1	(Yes = 1; No = 0)
Med. Pay. Expense				
(Public Insurance = 1)	78.74%	0	1	(Self-Pay = 0)
Community Care service (Yes = 1)	3.62%	0	1	(Not Provided = 0)

SD, standard deviation.

### 2.3 Statistical analysis

The software Stata 17 was utilized for data cleaning and analysis. Firstly, baseline values of different types of caregivers’ mental health burden were compared using *t*-tests and Pearson chi-square tests. Secondly, logistic regression models and ordered logistic regression models were employed to examine the correlation between caregiver-recipient relationship type and caregivers’ mental health burden (Xie, 2010). Subsample regression analysis was conducted to study different relationship types of caregivers. Lastly, to compare the relative influence of patient factors and family/community factors on caregivers’ mental health burden, the Sheaf coefficient method was used for post-estimation of logit regression models (Heise, 1972), utilizing the Stata commands proposed by Buis (2009).

For more information about the Sheaf coefficient method (Liu, 2015), which begins with the binomial logit model as shown in Eq. (1):


(1) $$\mathrm{logit}\frac{P}{1-P}=a+\overset{I}{\underset{i=1}{\sum }}\beta_{i}X_{i}+\overset{J}{\underset{j=1}{\sum }}\beta_{j}X_{j}+\overset{K}{\underset{k=1}{\sum }}\beta_{k}X_{k}+\varepsilon$$

$X_{i}$ represents a set of independent variables related to patient factors, $X_{j}$ represents a vector of independent variables related to family conditions, and $X_{k}$ represents a matrix of other control variables in the model. To compare the effects between patient factors and family conditions, the Sheaf coefficients method was introduced to the modeling process. Supposing the presence of a latent variable for patient factors ( $\eta_{a}$ , Eq. (2)) and a latent variable for family conditions ( $\eta_{b}$ , Eq. (3)), which are outcome variables of a series of independent variables $X_{i}$ related to patient factors and $X_{j}$ related to family conditions:


(2) $$\eta_{a}=c_{1}+\overset{I}{\underset{i}{\sum }}Z_{i}X_{i}$$

(3) $$\eta_{b}=c_{2}+\overset{I}{\underset{j}{\sum }}Z_{j}X_{j}$$

Eq. (1) is rewrote as follows:


(4) $$\mathrm{logit}\frac{P}{1-P}=a+\lambda_{1}\eta_{a}+\lambda_{2}\eta_{b}+\overset{K}{\underset{k=1}{\sum }}\beta_{k}X_{k}+\varepsilon$$

In fact, Eq. (4) is merely an alternative representation of Eq. (1), and it serves as an estimation performed after fitting model (Vigo et al., 2016). The objective of post-estimation is to simultaneously select two sets of parameters, $Z_{i}$ and $Z_{j}$ , such that the standard deviation of both $\eta_{a}$ and $\eta_{b}$ is set to 1. This ensures that the effects of the two cluster variables, $\eta_{a}$ and $\eta_{b}$ , are comparable.

## 3 Results

### 3.1 Sample characteristics

**Figure 1:**
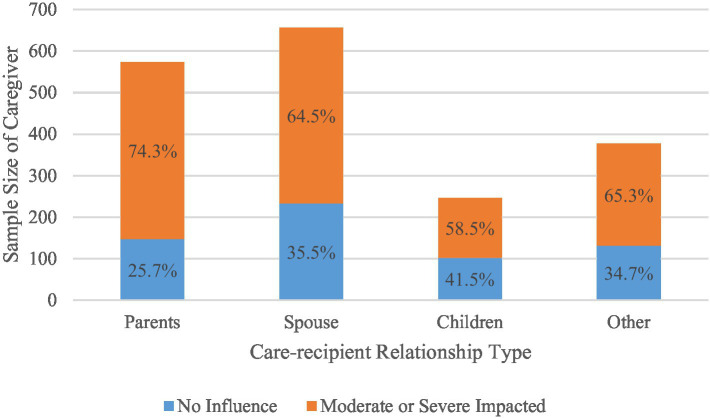
Care-recipient relationship type and caregivers’ mental health burden.

Results in Table 1 show that 66.9% of caregivers of schizophrenia patients in Beijing reported being moderately or severely impacted by their caregiving work. When utilizing the same measurement as a continuous variable, the mean score of caregiver’s mental health burden in China is 1.88, which is much higher than that (0.79 or 0.99) in India (Lasebikan and Ayinde, 2013; Swain et al., 2017). This result is in line with the findings of Chen’s survey in Beijing, where approximately 70% of respondents reported experiencing mental stress burden (Chen et al., 2019). Additionally, a total of 1,853 (62.9%) out of 2,993 schizophrenia caregivers in Beijing were included in current study, comprising 31% parental caregivers, 35.4% spousal caregivers, 13.3% adult-child caregivers, and 20.3% other caregivers. Furthermore, as depicted in Figure 1, 74.3% of parental caregivers reported being moderately or severely impacted by their caregiving responsibilities, while 64.5% of spousal caregivers, 58.5% of adult-child caregivers, and 65.3% of other caregivers reported similar experiences. These findings indicate that most caregivers of schizophrenia patients in China endure a heavy mental health burden.

In terms of caregivers, the average education duration was 9.96 years, which is higher than the national average year (9.91 years) of schooling for people over the age of 15 in China, presented by the seventh Chinese census conducted in 2020 (Akimov et al., 2021). Additionally, 85.6% of caregivers reported having a strong ability to care for their recipients. As for the care recipients, 53.86% were female, with an average age of 59.3 years. Moreover, 65% held disability certification, 93.42% were undergoing hospital treatment, and the mean duration of schizophrenia illness was 9.3 years. Regarding family and community conditions, the results reveal that 85% of caregivers were cohabiting with their care recipients. The average unweighted family income was 17,306 yuan, while the average percentage of household expenditure on medical expenses was 28%. Only 3.78% of families could afford market care services. Furthermore, public medical insurance was accessible to 78.74% of families, but at the community level, only 3.62% of families had received community care services.

### 3.2 Correlation between the caregiver-recipient relationship type and caregiver’s mental health

Table 2 displays the logistic regression model results regarding the impact of the relationship type between schizophrenia patients and caregivers on the caregiver’s mental health burden. Model 1 serves as the baseline model, indicating that compared to the spouse caregiver group, parent caregivers have significantly experienced more burden while caring for their family member with schizophrenia. Conversely, children acting as caregivers have experienced significantly less burden than the reference group, while other relation types show no significant differences from the spouse relationship type.

**Table 2:** Logistic regression models on the impact of care-recipient relationship type on mental health burden of schizophrenia patients’ caregivers in Beijing.

	(1)	(2)	(3)	(4)	(5)	(6)
Variables	Relation	+Care giver	+Patients	+Family	+Community	Full model
Relation (ref. Spouse)						
Parents	0.47^***^	0.46^***^	0.43^***^	0.48^***^	0.45^***^	0.43^***^
Children	−0.25^*^	−0.30^*^	−0.08	−0.24	−0.25	−0.16
Other	0.03	−0.02	0.06	0.02	0.02	−0.02
Care giver						
Education		0.01				0.03^**^
Self-rated ability		−0.35^**^				−0.29^*^
Patients						
Gender (ref. female)			0.31^***^			0.30^***^
Age/10			0.10^**^			0.08^*^
Disability Certif.			0.04			0.13
Under treatment			0.46^**^			0.51^**^
Time/10			0.31^***^			0.30^**^
Time/102			−0.07^***^			−0.07^***^
Family and community						
Living (ref. Live together)				−0.06		−0.03
Family income (log)				−0.04		−0.03
Consumption on Med.(log)				0.99***		0.93^***^
Consumption on care service				0.88***		0.85^***^
Med. Pay. Expense					−0.33^***^	−0.34^**^
Community care service					0.35	0.33
Constant	0.60^***^	0.77^***^	−0.76^**^	0.71	0.86^***^	−0.41
Observations	1,853	1,853	1,853	1,853	1,853	1,853
Pseudo R-square	0.01	0.01	0.02	0.02	0.01	0.04

^***^p < 0.01, ^**^p < 0.05, ^*^p < 0.1.

In Model 2, with the inclusion of caregivers’ personal characteristics as controls, the results remain largely unchanged from Model 1. Transitioning to Model 3, where we additionally consider the personal characteristics of mental illness patients, the findings indicate that, compared to the reference group, parent caregivers continue to experience significantly higher burden from caregiving activities, while child caregivers and other relation types do not differ significantly from the reference group.

Models 4 and 5 further incorporate family economic factors and community factors, with Model 6 representing the full model. The influence of caregivers’ relationship types with schizophrenia patients on the caregiver’s mental health burden remains relatively consistent across these models. Regarding control variables, the findings suggest that medical insurance coverage significantly reduces caregiver’s mental health burden, while community care services do not have a statistically significant impact. Overall, the results demonstrate that, compared to spouse caregivers, parent caregivers experience greater mental health burden from caregiving activities.

### 3.3 The influence mechanism of care-recipient relationship type on the caregiver’s mental health burden of schizophrenia patients

In Table 3, we have segmented the samples into four distinct groups based on the caregiver’s relationship types with the mental health patients, aiming to investigate the influence of various factors on the mental health burden of caregivers for schizophrenia patients. Model 1 reveals that, for parent caregivers, personal education significantly impacts their mental health burden. Additionally, male patients and whether they are undergoing treatment significantly contribute to their caregivers’ mental health burden. However, family and community factors do not show significant impacts on caregivers’ mental health burden.

**Table 3:** Logistic regression results across different relation types.

	(1)	(2)	(3)	(4)
Variables	Parents	Spouses	Children	Others
Care Giver				
Education	0.05^**^	0.02	−0.06	0.02
Self-rated ability	−0.17	−0.52^*^	−0.39	−0.09
Patients				
Gender (ref. female)	0.53^***^	0.30^*^	0.16	0.14
Age/10	−0.02	0.19^**^	−0.12	0.17^*^
Disability Certif.	0.14	0.31^*^	−0.51^*^	−0.01
Under treatment	0.74^**^	0.94^**^	0.13	0.25
Time/10	0.24	0.11	0.25	0.43^*^
Time/102	0.02	−0.05	−0.06	−0.08
Family and community				
Living (ref. Live together)	0.46	0.09	−0.08	−0.27
Family Income (log)	−0.08	−0.02	−0.11	0.07
Consumption on Med.(log)	0.66	1.12^***^	1.31^*^	0.69
Consumption on care service	−0.30	2.03^*^	0.71	1.26
Med. Pay. Expense	−0.24	−0.44^*^	−0.47	−0.23
Community care SERVICE	0.31	0.31	−0.26	0.71
Constant	−0.13	−1.33	3.18^**^	−1.67
Observations	573	657	246	377
Pseudo R-square	0.048	0.056	0.057	0.046

^***^p < 0.01, ^**^p < 0.05, ^*^p < 0.1.

Results in model 2 demonstrate that spouse caregivers’ self-rated ability significantly reduces their mental health burden. Furthermore, patients’ gender, age, disability certification, and treatment status also impact the mental health burden of spouse caregivers. Family and community factors, particularly expenditure on medical care and care services, significantly increase the mental health burden of spouse caregivers. Conversely, medical payment expenses significantly decrease their mental health burden. Models 3 and 4 present the findings for children and other relation types. Caregivers’ personal characteristics do not significantly impact their mental health burden in these groups. However, some patient characteristics and family/community conditions have a significant impact on the mental health burden of child caregivers. For other relation types, only schizophrenia patients’ personal characteristics significantly affect their mental health burden, particularly factors related to timing such as patient age and duration of illness.

Lastly, to compare the differences in various influencing factors on the mental health burden of caregivers across different relationship types, we employed the Sheaf coefficient method. The method allows us to compare the impact of patients’ ability factors and family economic factors on caregivers’ mental health burden. Results presented in Table 4 indicate that, for the full sample, the odds ratio is 4.53 (=e^1.51^), indicating that caregivers’ mental health burden primarily stems from family economic factors compared to patients’ ability factors.

**Table 4:** Comparison results based on the sheaf coefficient method.

	Full sample	Different relations			
		Parents	Spouses	Children	Others
I. Patients’ ability factors	0.15	0.24	0.19	0.18	0.19
II. Family or economic factors	0.22	0.18	0.28	0.26	0.23
Relative ratio (II/I)	1.51	0.77	1.43	1.45	1.23
Sample	1853	573	657	246	377

For parent caregivers, patients’ ability factors primarily contribute to their mental health burden. This suggests that most parent caregivers may lack the physical capabilities needed to provide optimal care for their adult child with schizophrenia. In contrast, for spouses and other relationship types, family economic factors emerge as the primary influencing factors. Spousal caregivers, who constitute the main caregiving group for schizophrenia patients in present Chinese society, bear a heavier mental and economic burden compared to other caregiving groups. Regarding children as caregivers, although family economic factors have a slightly higher relative impact compared to patients’ ability factors with an odds ratio of 4.24, their mental health burden is relatively small, as indicated by previous analysis results. Similarly, for caregivers in other relationship types, their mental health burden is primarily attributed to family economic factors.

## 4 Discussion

Findings of this study underscore the association between care-recipient relationship type and mental health burden experienced by caregivers of schizophrenia patients, with the influencing mechanism varying depending on the nature of the relationship. Our research, conducted among caregivers of schizophrenia patients in Beijing, China, highlights a notably high level of mental health burden, with 66.9% reporting moderate or severe impacts from their caregiving responsibilities. Schizophrenia, characterized by a disconnection between thoughts, emotions, and behaviors, often leads to withdrawal from reality and personal relationships. Caregivers of individuals with schizophrenia face significant challenges beyond ordinary imagination, navigating between cultural beliefs and modern treatment options to find a balance that aligns with their family’s preferences and values (Behrendt et al., 2019; Peng et al., 2019).

Findings reveal that parents and spouses serving as primary caregivers for schizophrenia patients in Beijing encounter a considerable burden of mental stress, with 74.3 and 64.5%, respectively, reporting moderate or severe impact from caregiving activities. Traditional Chinese values, emphasizing family cohesion and filial piety, likely influence caregiving dynamics for individuals with schizophrenia (Yan, 2023). Consequently, parents and spouses often bear the brunt of caregiving responsibilities, leading to heightened pressure and stress. While the distinction in burden levels between children and spousal caregivers lacked statistical significance, children generally reported lower burden levels than spousal caregivers. Similarly, caregivers in other relationship categories also indicated lower burden levels compared to spousal caregivers.

The present results indicate that the relationship type between caregivers and schizophrenia patients may have varying impacts on caregivers’ mental health burden, and the sources of this burden differ as well. These findings underscore the importance of studying the effects of care-recipient relationship type on caregivers’ mental health burden, particularly in the context of China’s market transition period. The lack of public care services has forced individuals to rely on assistance from within their families, resulting in family informal caregivers bearing a disproportionate amount of pressure that ideally should be shared by society.

The factors influencing caregivers’ mental health burden varied depending on the relationship type with schizophrenia patients. For many families of Chinese schizophrenia patients, the primary source of mental health burden for caregivers is not the patients themselves, but rather the responsibility of meeting caregiving duties while also sustaining the family’s livelihood. Parent caregivers typically experience mental health burden stemming from the personal factors of schizophrenia patients, whereas spousal caregivers or children as caregivers face socioeconomic factors, particularly economic burdens. Healthcare costs can pose significant challenges for Chinese families, especially when managing chronic conditions like schizophrenia. Out-of-pocket expenses for medications, therapy, and hospitalizations may strain household finances, particularly for low-income families or those lacking adequate insurance coverage.

Our results also indicate that only a small proportion of families can afford market-based care services, and most communities lack specific services for individuals with schizophrenia. In Beijing, various policies and measures have been implemented to support minority individuals, including schizophrenia patients, such as social, economic, medical, educational, and environmental support (Chen et al., 2019). Formal support programs could help reduce caregiver burden by improving caregivers’ stress management, coping skills, and access to informal sources of social support (Kamil and Velligan, 2019). However, there remains an insufficient provision of public services, particularly mental health support, for caregivers of mental health patients. Despite China’s progress in expanding mental health services, there is still a shortage of community-based support programs and resources tailored to caregivers of individuals with schizophrenia. This lack of support infrastructure can leave caregivers feeling overwhelmed and under supported.

The implication of this research primarily focuses on mitigating caregiver burden. Mental stress affects not only caregivers of schizophrenic patients but also those caring for individuals with other serious illnesses or elderly family members, highlighting the need for public sector attention to provide them with essential social and economic support. It is crucial to emphasize the value of reciprocity between caregivers and their mentally ill family members (Horwitz et al., 1996). The reciprocity norm may limit support for schizophrenia patients who cannot reciprocate. Despite this, family members still provide support, as family reciprocity does not need equal, direct, or immediate exchanges like economic transactions do. Instead, it is seen as a lifelong process where current support can reciprocate past support or anticipate future support. Additionally, the long-term care insurance, which is being piloted across the country (Feng et al., 2020), should provide more financial support for patients with schizophrenia and their caregivers. By sharing the economic burden of schizophrenia, the quality of life for schizophrenia patients will be enhanced and the economic and mental health burden on their caregivers will be alleviated. Moreover, humanistic care for caregivers of patients with schizophrenia should be enhanced, as long-term caregiving activities can often lead to mental burnout in these individuals, thereby negatively impacting the overall well-being of schizophrenia patients. As an old Chinese saying goes, “A parent of prolonged illness finds no dutiful children at the beside,” underscoring the importance of alleviating caregivers’ mental and economic burdens through effective system design (Sun et al., 2021). This is essential for elderly schizophrenia patients to enjoy their later years with dignity.

To our knowledge, this is the first study to utilize large-scale survey data to explore the correlation and influencing mechanisms between the care-recipient relationship type and the caregiver’s mental health burden of schizophrenia patients in China. However, this study also has several limitations. Firstly, due to its cross-sectional design and convenience sampling method, the results may not be generalizable to a broader population. Secondly, not all schizophrenia patients require or receive care from others; only those with severe symptoms or better economic circumstances do, potentially introducing selection bias into the sample data. Thirdly, the measurement of the binary format dependent variable may limit the rigor to capture varying levels of stress. Lastly, while other protective factors such as psychotherapy or counseling interventions could potentially reduce the mental health burden of caregivers of psychiatric patients, the lack of relevant measurements in the questionnaire makes it difficult to investigate these factors in this paper. Addressing these limitations will be imperative for future research endeavors.

## 5 Conclusion

This study indicates that care-recipient relationship type significantly influences mental health burden of caregivers among schizophrenia patients in Beijing, China. Both patient factors and family economic conditions affect caregivers’ mental health burden, with parental caregivers primarily affected by patients’ illness conditions, while spousal caregivers’ burden mainly stems from family economic conditions. Moreover, compared to spousal caregivers, the mental health burden of caregivers in the children, sibling, or other relationship categories experienced a comparatively lower mental health burden. Caregivers’ mental health, particularly for those caring for schizophrenia patients, is an important public concern. In China, the diverse types of care-recipient relationship lead to varying degrees of mental health burden among caregivers of schizophrenia patients. As a society deeply influenced by Confucian culture, people should uphold the culture of reciprocal relationships based on family affection. Besides, attention should be given to constructing public social services and providing humanistic care for caregivers of schizophrenia patients. This will enhance the quality of life for schizophrenia patients and alleviate the mental burden on caregivers.

## Data availability statement

The original contributions presented in the study are included in the article/supplementary material, further inquiries can be directed to the corresponding author.

## Ethics statement

The studies involving humans were approved by the Medical Ethics Committee of Capital Medical University in China. The studies were conducted in accordance with the local legislation and institutional requirements. The participants provided their written informed consent to participate in this study.

## Author contributions

YZ: Writing – original draft. MY: Writing – review & editing.
